# Impact of parental body mass index at diagnosis on obesity in survivors of pediatric craniopharyngioma

**DOI:** 10.1530/EC-24-0126

**Published:** 2024-07-17

**Authors:** Julia Beckhaus, Maria Eveslage, Brigitte Bison, Carsten Friedrich, Hermann L Müller

**Affiliations:** 1 Department of Pediatrics and Pediatric Hematology/Oncology, University Children’s Hospital, Carl von Ossietzky Universität, Klinikum Oldenburg AöR, Oldenburg, Germany; 2 Division of Epidemiology and Biometry, Carl von Ossietzky Universität, Oldenburg, Germany; 3 Institute of Biostatistics and Clinical Research, University of Münster, Münster, Germany; 4 Diagnostic and Interventional Neuroradiology, Faculty of Medicine, University of Augsburg, Augsburg, Germany

**Keywords:** craniopharyngioma, obesity, survivorship, parents, BMI

## Abstract

**Objective:**

It is well known that both genetic background and lifestyle influence the development of ‘general’ obesity. However, the role of parental body mass index (BMI) on the development of obesity in long-term survivors of childhood-onset craniopharyngioma (CP) is not well understood. This study analyzed the correlation of patients’ BMI at diagnosis and last visit and parental BMI at CP diagnosis and further explored potential risk factors for obesity in CP patients.

**Design:**

This is a registry-based retrospective cohort study.

**Methods:**

In total,291 CP patients and their parents recruited in the German KRANIOPHARYNGEOM studies were included. Correlations between patient’s BMI SDS at CP diagnosis and last visit and parental BMI at CP diagnosis were analyzed. The associations between hypothalamic damage, maternal/paternal BMI and CP patients’ obesity at last visit were analyzed by multivariable logistic regression.

**Results:**

At follow-up, 52% of CP patients developed obesity (BMI > 3SDS). Patient’s BMI SDS at last visit was moderately correlated with BMI-SDS at CP diagnosis (*r* = 0.48, 95% CI: 0.38–0.58, *P* < 0.001), and also with maternal BMI at diagnosis (*r* = 0.28, 95% CI: 0.17–0.38, *P* < 0.001) and paternal BMI at diagnosis (*r* = 0.3, 95% CI: 0.19–0.41, *P* < 0.001). However, the contributing role of parental BMI to the pathogenesis of obesity was small compared to the impact of hypothalamic damage.

**Conclusion:**

We conclude that besides hypothalamic damage, parental disposition for obesity is associated with the development of obesity in patients after CP. Our results indicate that also the family situation could have an influence on the development of obesity after CP and might be a therapeutic target.

**Significance statement:**

Survivors of childhood-onset craniopharyngioma are at risk of developing morbid obesity. So far, patients with posterior hypothalamic involvement and lesion were identified as a high risk group. With this study, the influence of parental body mass index on the risk of obesity was investigated. Patient’s body-mass-index at last visit was correlated with maternal and paternal body mass index at diagnosis. With increasing maternal or paternal body mass index, the likelihood of obesity in individuals with CP increased. Nevertheless, the parents’ weight had only a small effect on the development of patients’ obesity compared to hypothalamic damage.

## Introduction

Craniopharyngiomas (CP) are rare embryonic malformations, which arise from ectoblastic remnants of Rathke’s pouch. Thus, CP can be found anywhere along the path of development of Rathke’s pouch in hypothalamic and pituitary regions, which are important for endocrine regulation and satiety modulation (1). Adamantinomatous CP are the most common intracranial tumors of non-glial origin in the pediatric population (2). Worldwide, the incidence of CP is estimated at 0.5 to 2 new cases per million persons per year (3). The peak incidence is at age 5 to 9 years but CP can occur at any age including infancy and neonatal period (3, 4).

Despite the low grade of histological malignancy (WHO grade 1) of CP, their intimate association with hypothalamic structures frequently predispose patients with these tumors to several adverse sequelae, which can be summarized by the so-called hypothalamic syndrome (5). In a random sample of the German KRANIOPHARYNGEOM 2007 study and KRANIOPHARYNGEOM Registry 2019, 36 out of 60 (60%) of the included patients were classified with hypothalamic syndrome (6). Obesity was present in 35% (*n* = 21) of these patients (6). Hypothalamic syndrome is also characterized by abnormal eating behavior such as hyperphagia (5). Patients with hypothalamic lesions often present with higher food inattention, lower satiety and higher restrained eating behavior (7). However, hyperphagia is not always present in patients with CP, reflecting that in some patients specific hypothalamic nuclei are still intact after treatment (2). A damaged mediobasal hypothalamus leads to dysfunction of proopiomelanocortin and agouti-related peptide-neuropeptide Y neurons, a disruption of central and peripheral hormones and therefore abnormal eating behavior (8). In addition to abnormal eating patterns, patients with CP have a reduced basal metabolic rate, which may contribute to the development of obesity (9, 10).

It is well known that both genetic backgroup and socioeconomic and lifestyle factors influence the development of ‘general’ obesity not caused by sellar masses and deficiency of the hypothalamic-pituitary axes. According to the German Health Interview and Examination Survey for Children and Adolescents (KiGGS) 2006, parental overweight was, besides socioeconomic factors, a main risk factor for obesity in children and adolescents (3–17 years) of the general population (11). Maternal overweight and paternal overweight were associated with obesity in children of the general population (11). However, in patients with childhood-onset CP, the association of parental body mass index (BMI) and obesity after treatment is not well understood.

In our study, body weight, body height, and BMI in patients and their parents at the time of CP diagnosis were analyzed. Furthermore, we assessed the correlation of the patient’s BMI standard deviation score (SDS) at CP diagnosis and at follow-up with the parental BMI at CP diagnosis and the association of parental BMI and hypothalamic damage with obesity at last visit during follow-up in childhood-onset CP survivors.

## Patients and methods

### Study design and participants

709 (351 (49.5) females/358 (50.5) males) diagnosed with adamantinomatous CP (median age at last visit: 18.2 years, ranging from 0.4 to 45.3 years) were recruited between 1999 and 2021 in the clinical trials/registries HIT-Endo, KRANIOPHARYNGEOM 2000 (clinical trial no. NCT00258453), KRANIOPHARYNGEOM 2007 (clinical trial no. NCT01272622) (12) and in the KRANIOPHARYNGEOM Registry 2019 (clinical trial no. NCT04158284) and prospectively observed with a median follow-up interval of 8.37 years (ranging from 0.04 to 38.87 years). Eligibility criteria of the studies and registries were: adamantinomatous CP diagnosis confirmed by central pathological review, age <18 years at CP diagnosis, residing in Germany, Austria, Switzerland or Belgium.

The following analyses included a subset of the 291 patients, with available data on patient BMI and at least one parental BMI at CP diagnosis, a minimal follow-up of 1 year after CP diagnosis and information on patient BMI SDS at last visit.

### Neuroimaging

According to the KRANIOPHARYNGEOM 2007 and KRANIOPHARYNGEOM Registry 2019 protocols (13, 14, 15), cranial magnetic resonance imaging (MRI) were performed at the time points of CP diagnosis and prospectively at 3-months intervals during the first year of follow-up after CP diagnosis. A neuroradiologist (BB), blinded for clinical details, evaluated the hypothalamic involvement before surgery (HI), the position of the tumor, the extent of surgical removal, and any resulting surgical hypothalamic lesions (HL). The HI of the CP was classified into distinct grades: grade 0 indicated no observable HI on preoperative MRIs, grade I denoted HI of anterior parts of the hypothalamus without affecting the mammillary bodies (MB) and the posterior structures, and grade II indicated HI of both the anterior and posterior parts of the hypothalamus, including the anterior area, MB, and structures behind the MB. After surgery, HL were classified into three grades as mentioned above: grade 0 HL indicated no detectable HL on postoperative MRIs; grade I HL indicated lesioned anterior hypothalamic structures not involving MB, and grade II described involving anterior hypothalamic areas, MB and hypothalamic structures dorsal of MB. The detailed description of neuroradiological assessment was published elsewhere (13, 14).

For patients recruited in the studies HIT-Endo and KRANIOPHARYNGEOM 2000, the assessments of HI and HL were based on medical records, if no MRIs were available.

### Clinical parameters

In all patients, clinical and demographic parameters were analysed based on the protocols of the trials HIT-Endo, KRANIOPHARYNGEOM 2000/2007 and KRANIOPHARYNGEOM Registry 2019 (16). Body weight, body height, and BMI of the patients were measured at CP diagnosis and at last visit (2019–2022). BMI (*w*/*h*^2^; *w* = weight (kg), *h* = height (m)) was calculated for the parents at CP diagnosis. For each patient, BMI was calculated as SDS according to the age-related references of Rolland-Cachera *et al.* at CP diagnosis and at last visit (17). Obesity at last visit was defined as a BMI ≥ 3 SDS. For the parents, BMI (*w*/*h*^2^; *w* = weight (kg), *h* = height (m)) was calculated at CP diagnosis and categorized in overweight (>25 kg/m²) or no overweight (≤25 kg/m²) based on self-reported weight and height.

### Statistical analysis

Statistical analyses were performed using R software, version 4.2.1. Data are displayed as median (range) or frequency (percent). Pearson’s correlation coefficient was determined for normally distributed continuous variables in order to assess the degree of linear correlation. Multivariable logistic regression was applied to assess the association of hypothalamic damage and BMI on patient’s obesity at last visit. Results are presented as odds ratios and corresponding 95% CIs. Cases with missing data were excluded in terms of complete case analyses. Two-sided *P*-values are given and a *P*-value ≤ 5% is considered statistically noticeable. However, all analyses were intended to be exploratory in nature and no adjustment for multiple testing was applied.

### Ethical considerations

The study protocols of HIT-Endo, KRANIOPHARYNGEOM 2000/2007 were reviewed and accepted by the ethical committee of the University of Würzburg, Germany (140/99; 94/06); the protocol of KRANIOPHARYNGEOM Registry 2019 by the ethical committee of the Carl von Ossietzky Universität Oldenburg, Germany (2019-046). The studies comply with the Declaration of Helsinki. All patients were informed according to the study protocols and gave informed consent.

## Results

In a subset of 291 of 709 CP patients (41.1%), data on patient and parental BMI at CP diagnosis and on patient BMI development with a minimal follow-up of 1 year were available for analyses (median follow-up interval: 9.38 years; range: 1.05–33.4 years). Table 1 summarizes patient’s characteristics according to maternal BMI (≤25 vs >25 kg/m²) at CP diagnosis. Table 2 shows patient’s characteristics according to paternal BMI (≤25 vs >25 kg/m²) at CP diagnosis. Sex was almost equally distributed in the overall group (153 (52.6%) female patients). Median age at CP diagnosis was 9.49 years (range: 0.01–17.9 years) and median age at last visit was 19.1 years (range: 1.97–41.7 years). Children of parents with overweight (>25 kg/m²) at the time point of CP diagnosis, presented with a higher median BMI SDS at CP diagnosis (children with maternal overweight: BMI at diagnosis +1.40 SDS (range: −2.00 to +9.90 SDS); children with paternal overweight: BMI at CP diagnosis +0.95 SDS (range: −3.23 to +9.90 SDS)). Regarding hypothalamic damage, 34% (*n* = 99) of the overall group had no HI or HL (Table 1). Taking surgical HL into account, 62 (21.3%) of the patients with overweight mothers and 57 (20.6%) of the patients with overweight fathers were classified with HI grade I/II and HL grade II. In the maternal resp. paternal non-overweight groups, no surgical HL was present in 66 (22.7%) resp. 64 (23.1%) patients with presurgical HI grade I or II.

**Table 1:** Baseline characteristics of patients with childhood-onset craniopharyngioma (CP) according to maternal body mass index (BMI) at craniopharyngioma diagnosis.

	Maternal BMI ≤ 25 kg/m²	Maternal BMI > 25 kg/m²	Overall
	(*n* = 163)	(*n* = 128)	(*n* = 291)
Sex			
Male	76 (46.6%)	62 (48.4%)	138 (47.4%)
Female	87 (53.4%)	66 (51.6%)	153 (52.6%)
Age at diagnosis (years)			
Median (minimum, maximum)	9.48 (0.01, 17.9)	9.53 (0.05, 17.5)	9.49 (0.01, 17.9)
Age at last visit (years)			
Median (minimum, maximum)	18.6 (1.97, 41.0)	19.5 (5.34, 41.7)	19.1 (1.97, 41.7)
Follow-up time (y)			
Median (minimum, maximum)	8.53 (1.08, 31.7)	9.95 (1.05, 33.4)	9.38 (1.05, 33.4)
BMI SDS at CP diagnosis (17)			
Median (minimum, maximum)	0.400 (−3.73, 6.40)	1.40 (−2.00, 9.90)	0.725 (−3.73, 9.90)
Missing data	22 (13.5%)	15 (11.7%)	37 (12.7%)
Grade of HI/HL (13, 14)			
HI and HL grade 0	56 (34.4%)	43 (33.6%)	99 (34.0%)
HI grade I/II and HL grade I	38 (23.3%)	22 (17.2%)	60 (20.6%)
HI grade I/II and HL grade II	28 (17.2%)	34 (26.6%)	62 (21.3%)
HI grade I/II and HL grade 0	38 (23.3%)	28 (21.9%)	66 (22.7%)
Missing data	3 (1.8%)	1 (0.8%)	4 (1.4%)
Visual impairment at CP diagnosis	16 (9.8%)	18 (14.1%)	34 (11.7%)
Missing data	133 (81.6%)	97 (75.8%)	230 (79.0%)

BMI, body mass index; CP, craniopharyngioma; HI, presurgical hypothalamic involvement; HL, surgical hypothalamic lesion; SDS, standard deviation score.

**Table 2:** Baseline characteristics of patients with childhood-onset craniopharyngioma (CP) according to paternal body mass index (BMI) at craniopharyngioma diagnosis.

	Paternal BMI ≤ 25 kg/m²	Paternal BMI > 25 kg/m²	Overall
	(*n* = 114)	(*n* = 163)	(*n* = 277)
Sex			
Male	56 (49.1%)	77 (47.2%)	133 (48.0%)
Female	58 (50.9%)	86 (52.8%)	144 (52.0%)
Age at CP diagnosis (years)			
Median (minimum, maximum)	10.1 (0.01, 17.9)	8.49 (0.05, 17.6)	9.48 (0.01, 17.9)
Age at last visit (years)			
Median (minimum, maximum)	19.4 (1.97, 41.7)	18.5 (5.34, 41.0)	19.0 (1.97, 41.7)
Follow-up time (years)			
Median (minimum, maximum)	9.56 (1.10, 31.7)	8.95 (1.05, 33.4)	9.38 (1.05, 33.4)
BMI SDS at CP diagnosis (17)			
Median (minimum, maximum)	0.420 (−3.73, 7.55)	0.955 (−3.23, 9.90)	0.700 (−3.73, 9.90)
Missing data	13 (11.4%)	23 (14.1%)	36 (13.0%)
Grade of HI/HL (13, 14)			
HI and HL grade 0	39 (34.2%)	56 (34.4%)	95 (34.3%)
HI grade I/II and HL grade I	33 (28.9%)	25 (15.3%)	58 (20.9%)
HI grade I/II and HL grade II	18 (15.8%)	39 (23.9%)	57 (20.6%)
HI grade I/II and HL grade 0	22 (19.3%)	42 (25.8%)	64 (23.1%)
Missing data	2 (1.8%)	1 (0.6%)	3 (1.1%)
Visual impairment at CP diagnosis	9 (7.9%)	21 (12.9%)	30 (10.8%)
Missing data	95 (83.3%)	125 (76.7%)	220 (79.4%)

BMI, body mass index; CP, craniopharyngioma; HI, presurgical hypothalamic involvement; HL, surgical hypothalamic lesion; SDS, standard deviation score.

**Figure 1:**
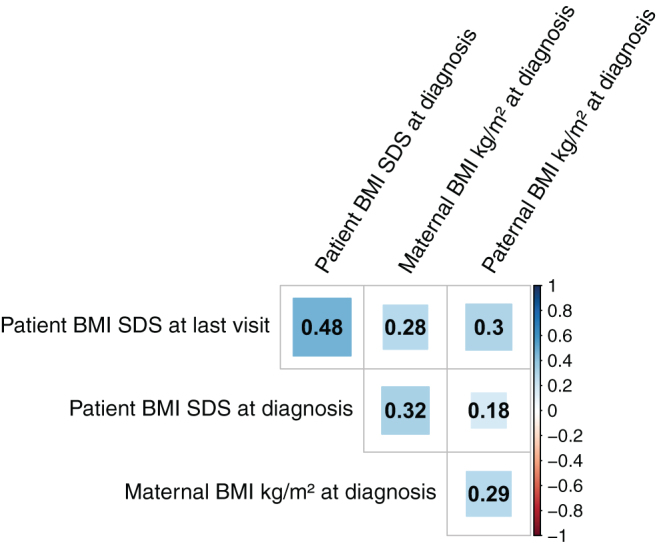
Pearson’s correlation plot between maternal body mass index (BMI), paternal BMI at the time of craniopharyngioma (CP) diagnosis and patient BMI SDS at the time of diagnosis and at last visit during follow-up.

Figure 1 illustrates the Pearson’s correlation coefficients between patient’s BMI SDS at diagnosis, at last visit, maternal BMI at diagnosis and paternal BMI at diagnosis. All BMI values were positively correlated. Patient’s BMI SDS at last visit was moderately correlated with patient’s BMI SDS at diagnosis (*r* = 0.48, 95% CI: 0.38–0.58, *P* < 0.001), also with maternal BMI at diagnosis (*r* = 0.28, 95% CI: 0.17–0.38, *P* < 0.001) and paternal BMI at diagnosis (*r* = 0.3, 95% CI: 0.19–0.41, *P* < 0.001). At CP diagnosis, the correlation between patient’s BMI SDS and the maternal BMI was also moderate (*r* = 0.32, 95% CI: 0.21–0.43, *P* < 0.001). Between patient’s BMI SDS and the paternal BMI at diagnosis, the correlation was weak (*r* = 0.18, 95% CI: 0.05–0.3, *P* = 0.006). Maternal BMI at diagnosis correlated moderately with paternal BMI at diagnosis (*r* = 0.29, 95% CI: 0.18–0.39, *P* < 0.001).

During a median follow-up interval of 9.38 years (range: 1.05–33.4 years), 151 of 291 patients (51.8%) developed obesity (BMI > 3.0 SDS). In univariable analyses, HI I/II and HL II were associated with increased odds for obesity (OR = 6.69, 95% CI: 3.24–14.65).

The odds of the outcome variable obesity at last visit increased by 1.43 (95% CI: 1.25–1.66) times for every unit increase in patient’s BMI SDS at diagnosis, 1.12 (95% CI: 1.06–1.18) times for every unit increase in maternal BMI and 1.13 (95% CI: 1.06–1.22) times for every unit increase in paternal BMI (Table 3).

**Table 3:** Results of univariable logistic regression of hypothalamic damage, patient’s BMI SDS at craniopharyngioma diagnosis, maternal BMI at diagnosis and paternal BMI at diagnosis on patient’s obesity at last visit.

Characteristics		Unadjusted OR	Lower limit 95% CI	Upper limit 95% CI
HI and HL (13, 14)	HI and HL grade 0	Reference		
	HI grade I/II and HL grade I	1.31	0.69	2.52
	HI grade I/II and HL grade II	6.69	3.24	14.65
	HI grade I/II and HL grade 0	1.61	0.86	3.03
Patient’s BMI SDS at CP diagnosis		1.43	1.25	1.66
Maternal BMI at CP diagnosis (kg/m²)		1.12	1.06	1.18
Paternal BMI at CP diagnosis (kg/m²)		1.13	1.06	1.22

BMI, body mass index; CP, craniopharyngioma; HI, presurgical hypothalamic involvement; HL, surgical hypothalamic lesion; OR, odds ratio.

**Figure 2:**
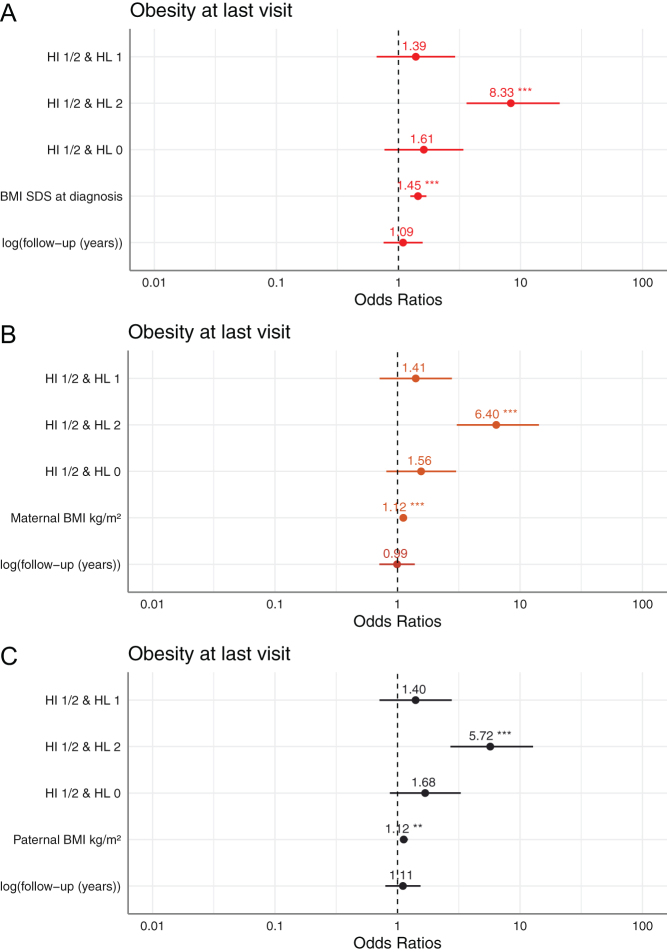
**A-C:**Forest plots of multivariable logistic regression models on craniopharyngioma (CP) patient’s obesity (BMI > 3 SDS) at last visit (*P ≤ 0.05; **P ≤ 0.01; ***P ≤ 0.001). Including the variables hypothalamic damage (reference HI/HL 0), patient’s BMI SDS at diagnosis and (log) follow-up. Including the variables hypothalamic damage (reference HI/HL 0), maternal BMI kg/m² at diagnosis and (log)follow-up. Including the variables hypothalamic damage (reference HI/HL 0), paternal BMI kg/m² at diagnosis and (log)follow-up.

After adjustment for follow-up, the odds ratios on patient’s obesity at last visit from three multivariable regression models for hypothalamic damage and patient’s BMI SDS at diagnosis, maternal BMI or paternal BMI are shown in Fig. 2A, B and C.

Posterior/anterior HI and HL (grade II) was a risk factor for obesity at last visit in all three models (Fig. 2). In the regression with paternal BMI, the OR on patient’s obesity was 5.72 (95% CI: 2.71–12.79), with maternal BMI (OR = 6.4, 95% CI: 3.05–14.26) and with patient’s BMI at diagnosis (OR = 8.33, 95% CI: 3.62–20.93) for HI grade I/II and HL grade II.

Patient’s BMI SDS at diagnosis was associated with obesity at last visit (OR = 1.44, 95% CI: 1.25–1.66) after adjustment for follow-up and hypothalamic damage. Maternal BMI at diagnosis was also associated with obesity at last visit (OR = 1.11, 95% CI: 1.06–1.18) in the multivariable analysis. The OR of paternal BMI for obesity at last visit, did also not change substantially after adjustment (OR = 1.12, 95% CI: 1.05–1.54) compared to the unadjusted OR. The odds of having obesity at last visit was 2.87 times higher (95% CI: 1.53–5.49) as a child of both overweight (>25 kg/m² BMI) parents compared to children of normal weight parents at CP diagnosis (Supplementary Table 1, see section on supplementary materials given at the end of this article).

Based on the multivariable logistic regression models presented in Fig. 2A, B and C, the probability for patient obesity at last visit was estimated. In Supplementary Fig. 1, the probability of obesity is shown according to different values of maternal BMI in four categories of hypothalamic damage, adjusted for follow-up. With increasing maternal BMI, the probability of having obesity as a patient with CP increases. The confidence intervals of the strata with anterior or no post-surgical HL overlapped, indicating no statistically noticeable differences between these groups. For patients with HI and posterior HL, the probability of developing obesity is increased, also for maternal normal weight (BMI: 20–25). With maternal BMI of 20, the probability of developing obesity was estimated at 69% (95% CI: 53–82%), with a BMI of 25 at 80% (95% CI: 67–88%) and with a BMI of 30 at 87% (95% CI: 77–93%) in patients with HI and posterior HL (grade II).

In Supplementary Fig. 2, the probability of obesity at last visit is shown according to different values of paternal BMI in four categories of hypothalamic damage, adjusted for follow-up. With increasing paternal BMI, the probability of developing obesity as a patient with CP increases. However, the confidence intervals for the hypothalamic damage groups overlapped, indicating no statistically noticeable differences between the groups. Regarding the group with HI and posterior HL, the probability of developing obesity with a paternal BMI of 20 was estimated at 62% (95% CI: 42–79%), with a paternal BMI of 25 at 75% (95% CI: 61–85%) and with a paternal BMI of 30 at 84% (95% CI: 73–91%).

## Discussion

Obesity in patients with childhood-onset CP is not only associated with hypothalamic lesions due to CP treatment but also with parental BMI. However, the contribution of parental degree of obesity was small compared to the role of hypothalamic damage. Disease- and/or treatment-associated lesions of posterior hypothalamic structures contribute as pathogenic mechanisms to the development of obesity after CP (18). Accordingly, hypothalamus-sparing treatment strategies are currently favored as the therapy of choice and best option for prevention or amelioration of hypothalamic obesity (13, 19). Treatment approaches for obesity as clinical manifestation of a hypothalamic syndrome are very limited (5).

Genetic predisposition, specific lifestyle factors, and socioeconomic status were frequently identified as risk factors for the development of ‘general obesity’ unrelated to sellar masses and hypothalamic syndrome (11). Previously, it was shown that maternal BMI at the time of CP diagnosis was associated with the development of severe obesity after CP in their offspring (20). In our current study, we replicate this finding on maternal BMI at CP diagnosis as a potential risk factor and furthermore elucidate the association of paternal BMI at CP diagnosis on the development of obesity in offspring with CP.

Based on our data, it is not possible to discriminate whether parental BMIs as observed risk factors for the development of obesity in CP patients reflect a genetic disposition or lifestyle condition, or a combination of both. Since maternal, paternal and patient’s BMI share the same risk factors the results cannot be interpreted causally. Nonetheless, our findings have important implications for treatment and prevention of severe hypothalamic obesity in CP patients.

In families presenting with the above-mentioned parental characteristics of high BMI, especially CP patients with further risk factors for obesity such as disease- and/or treatment-related lesions of posterior hypothalamic structures (HL grade II) should be monitored closely and interventions initiated early. Interventions should not only address the patient, but also the families need to be included in nutritional and physical exercise interventions (21). Early recognition of hypothalamic obesity and a patient-centered approach are necessary to intervene before a massive weight gain occurs (22). As suggested by van Roessel *et al.,* important etiologic factors for increased BMI at diagnosis of a childhood brain tumor include lifestyle and genetics (23). Their results implicate the importance of timely lifestyle interventions directly at diagnosis, although the management of hypothalamic obesity is complex (23).

In a recent survey in the USA, 106 caregivers of patients with CP were assessed regarding the child’s diagnosis, medication, lifestyle modifications and social function (21). 60 of 106 caregivers (57%) reported that their child had obesity and that they tried a variety of interventions for management of excess weight gain. The majority (92%) of patients living with obesity and CP attempted to restrict either calories or carbohydrates, with 31% and 69% reporting these measures as helpful, respectively. Additionally, 38% tried weight loss medications such as central stimulating agents, metformin, glucagon-like peptide 1 receptor (GLP1R) agonists, and topiramate, and 48% found at least one of these medications to be beneficial (21). The multifactorial etiology of hypothalamic obesity needs a multifactorial prevention and treatment, with early-onset lifestyle, physical activity interventions and effective medications, which help to regulate hunger attacks (23). Multiple previous studies on patients with childhood-onset CP have shown that obesity and the resulting body image have a crucial impact on quality of life of the long-term survivors (24, 25, 26).

Our study has certain limitations and strengths. We do not have data on further development of parental BMIs during long-term follow-up of their children suffering from CP. Additionally, data on familial disposition for obesity such as BMI of siblings were not available for analysis in our multicenter observational KRANIOPHARYNGEOM studies. Furthermore, the application of three different logistic regression models to assess the association of maternal and paternal BMI and patient’s obesity at last visit cannot fully reflect the causal relationships between parental and patient’s BMI. For example, it is not possible to estimate the effect of BMI at CP diagnosis while adjusting for parental BMI because BMI at diagnosis is associated both with BMI of the mother and the father (Fig. 1). The resulting odds ratios should therefore be interpreted with caution, since we cannot rule out an omitted variable bias. Data on specific lifestyle characteristics such as nutritional characteristics or socioeconomic status of the families were not available for analyses. Usually, the rareness of the disease results in certain limitations due to low cohort size. However, in our analyses the cohort size of 292 patients with this rare disease should be pointed out as a strength of our study. Nonetheless, the interpretability of the results of the multivariable logistic regression and the resulting probabilities is limited due to the wide confidence intervals.

Further research on survivors after CP should examine prospectively the patient’s but also the family’s situation in terms of anthropometric development but also their socioeconomic situation and quality of life (27, 28). Randomized-controlled intervention studies are required to examine the efficacy of physical and nutritional interventions and anti-obesity pharmaceutical agents in this specific population.

In conclusion, parental BMI has a small but contributing role to the risk of obesity of their children with CP. However, based on previous reports hypothalamus-sparing treatment strategies are recommended to prevent or ameliorate hypothalamic obesity due to lesions of posterior hypothalamic structures (13, 14, 18, 19, 24, 25, 26). Furthermore, familial disposition for obesity – clinically observed as high parental BMIs – should lead to an early intensification of interventions for obesity prevention and treatment.

## Supplementary Materials

Supplementary Material

## Declaration of interest

HLM has received reimbursement of participation fees for scientific meetings and continuing medical education events from the following companies: Ferring, Lilly, Pfizer, Sandoz/Hexal, Novo Nordisk, IPSEN, and Merck Serono. He has received reimbursement of travel expenses from Merck, Rhythm Pharmaceuticals, and lecture honoraria from Pfizer and Rhythm Pharmaceuticals. BB received honorary from Merck Serono for a continuing medical education lecture. The other authors declare that they have no conflict of interest that could be construed as prejudicing the impartiality of the study reported.

## Funding

This study was funded by grants (HLM, DKS2014.13; BB, DKS2018.02) of the German Childhood Cancer Foundation, Bonn, Germany.

## Author contributions

JB: conceptualization, methodology, formal analysis, writing – original draft and review and editing; ME: methodology, supervision, writing – review and editing; CF: supervision, writing – review and editing; BB: writing – review and editing; HLM: conceptualization, writing – original draft and review and editing, supervision, project administration, funding acquisition.

## Data availability

Restrictions apply to the availability of all data generated or analyzed during this study to preserve patient confidentiality. The corresponding author will on request detail the restrictions and any conditions under which access to some data may be provided.

## References

[1] Santagata SK-DB Komori T Müller HL Pietsch T. Adamantinomatous craniopharyngioma. In WHO Classification of Tumours, Central Nervous System Tumours, 5th ed., vol 6, pp. 393–396, Lyon, France: International Agency for Research on Cancer, 2021.

[2] Apps JR Muller HL Hankinson TC Yock TI & Martinez-Barbera JP. Contemporary biological insights and clinical management of craniopharyngioma. Endocrine Reviews 2023 44 518–538. (10.1210/endrev/bnac035) 36574377

[3] Muller HL. Craniopharyngioma. Endocrine Reviews 2014 35 513–543. (10.1210/er.2013-1115) 24467716

[4] Beckhaus J Boekhoff S Scheinemann K Schilling FH Fleischhack G Binder G Bison B Pietsch T Friedrich C & Müller HL. Perinatally diagnosed congenital craniopharyngiomas in the KRANIOPHARYNGEOM trials. Endocrine Connections 2023 12. (10.1530/EC-23-0294) PMC10692685 37878777

[5] Müller HL Tauber M Lawson EA Özyurt J Bison B Martinez-Barbera J-P Puget S Merchant TE & van Santen HM. Hypothalamic syndrome. Nature Reviews 2022 8 24. (10.1038/s41572-022-00351-z) 35449162

[6] van Santen HM van Schaik J van Roessel IMAA Beckhaus J Boekhoff S & Müller HL. Diagnostic criteria for the hypothalamic syndrome in childhood. European Journal of Endocrinology 2023 188 214–225. (10.1093/ejendo/lvad009) 36737045

[7] Lee M Park MJ Lee KH Kim JH Choi HJ & Kim YH. Obesity mechanism after hypothalamic damage: cohort analysis of neuroimaging, psychological, cognitive, and clinical phenotyping data. Frontiers in Endocrinology 2023 14 1114409. (10.3389/fendo.2023.1114409) 37056667 PMC10086156

[8] van Iersel L Brokke KE Adan RAH Bulthuis LCM van den Akker ELT & van Santen HM. Pathophysiology and individualized treatment of hypothalamic obesity following craniopharyngioma and other suprasellar tumors: a systematic review. Endocrine Reviews 2019 40 193–235. (10.1210/er.2018-00017) 30247642

[9] Shaikh MG Grundy RG & Kirk JMW. Reductions in basal metabolic rate and physical activity contribute to hypothalamic obesity. Journal of Clinical Endocrinology and Metabolism 2008 93 2588–2593. (10.1210/jc.2007-2672) 18413428

[10] Holmer H Pozarek G Wirfält E Popovic V Ekman B Björk J & Erfurth EM. Reduced energy expenditure and impaired feeding-related signals but not high energy intake reinforces hypothalamic obesity in adults with childhood onset craniopharyngioma. Journal of Clinical Endocrinology and Metabolism 2010 95 5395–5402. (10.1210/jc.2010-0993) 20826582

[11] Kleiser C Schaffrath Rosario A Mensink GBM Prinz-Langenohl R & Kurth BM. Potential determinants of obesity among children and adolescents in Germany: results from the cross-sectional KiGGS Study. BMC Public Health 2009 9 46. (10.1186/1471-2458-9-46) 19187531 PMC2642815

[12] Muller HL Gebhardt U Etavard-Gorris N Korenke E Warmuth-Metz M Kolb R Sorensen N & Calaminus G. Prognosis and sequela in patients with childhood craniopharyngioma -- results of HIT-ENDO and update on KRANIOPHARYNGEOM 2000. Klinische Padiatrie 2004 216 343–348. (10.1055/s-2004-832339) 15565549

[13] Muller HL Gebhardt U Teske C Faldum A Zwiener I Warmuth-Metz M Pietsch T Pohl F Sorensen N Calaminus G, *et al.* Post-operative hypothalamic lesions and obesity in childhood craniopharyngioma: results of the multinational prospective trial kraniopharyngeom 2000 after 3-year follow-up. European Journal of Endocrinology 2011 165 17–24. (10.1530/EJE-11-0158) 21490122

[14] Muller HL Gebhardt U Faldum A Warmuth-Metz M Pietsch T Pohl F Calaminus G Sorensen N & KRANIOPHARYNGEOM 2000 Study Committee. Xanthogranuloma, Rathke's cyst, and childhood craniopharyngioma: results of prospective multinational studies of children and adolescents with rare sellar malformations. Journal of Clinical Endocrinology and Metabolism 2012 97 3935–3943. (10.1210/jc.2012-2069) 22969141

[15] Warmuth-Metz M Gnekow AK Muller H & Solymosi L. Differential diagnosis of suprasellar tumors in children. Klinische Padiatrie 2004 216 323–330. (10.1055/s-2004-832358) 15565547

[16] Hoffmann A Warmth-Metz M Gebhardt U Pietsch T Pohl F Kortmann RD Calaminus G & Muller HL. Childhood craniopharyngioma - changes of treatment strategies in the trials kraniopharyngeom 2000/2007. Klinische Padiatrie 2014 226 161–168. (10.1055/s-0034-1368785) 24819386

[17] Rolland-Cachera MF Cole TJ Sempe M Tichet J Rossignol C & Charraud A. Body mass index variations: centiles from birth to 87 years. European Journal of Clinical Nutrition 1991 45 13–21. 1855495

[18] Bogusz A Boekhoff S Warmuth-Metz M Calaminus G Eveslage M & Muller HL. Posterior hypothalamus-sparing surgery improves outcome after childhood craniopharyngioma. Endocrine Connections 2019 8 481–492. (10.1530/EC-19-0074) 30925462 PMC6479199

[19] Elowe-Gruau E Beltrand J Brauner R Pinto G Samara-Boustani D Thalassinos C Busiah K Laborde K Boddaert N Zerah M, *et al.* Childhood craniopharyngioma: hypothalamus-sparing surgery decreases the risk of obesity. Journal of Clinical Endocrinology and Metabolism 2013 98 2376–2382. (10.1210/jc.2012-3928) 23633208

[20] Muller HL Bueb K Bartels U Roth C Harz K Graf N Korinthenberg R Bettendorf M Kuhl J Gutjahr P, *et al.* Obesity after childhood craniopharyngioma: German multicenter study on pre-operative risk factors and quality of life. Klinische Padiatrie 2001 213 244–249. (10.1055/s-2001-16855) 11528558

[21] Craven M Crowley JH Chiang L Kline C Malbari F Hocking MC & McCormack SE. A survey of patient-relevant outcomes in pediatric craniopharyngioma: focus on hypothalamic obesity. Frontiers in Endocrinology 2022 13 876770. (10.3389/fendo.2022.876770) 35615720 PMC9124861

[22] Shoemaker AH & Tamaroff J. Approach to the patient with hypothalamic obesity. Journal of Clinical Endocrinology and Metabolism 2023 108 1236–1242. (10.1210/clinem/dgac678) 36413492 PMC10306088

[23] van Roessel IMAA van Schaik J Meeteren AYNS Boot AM der Grinten HLC Clement SC van Iersel L Han KS van Trotsenburg ASP Vandertop WP, *et al.* Body mass index at diagnosis of a childhood brain tumor; a reflection of hypothalamic-pituitary dysfunction or lifestyle? Supportive Care in Cancer 2022 30 6093–6102. (10.1007/s00520-022-07031-4) 35416504 PMC9135856

[24] Sterkenburg AS Hoffmann A Gebhardt U Warmuth-Metz M Daubenbuchel AMM & Muller HL. Survival, hypothalamic obesity, and neuropsychological/psychosocial status after childhood-onset craniopharyngioma: newly reported long-term outcomes. Neuro-Oncology 2015 17 1029–1038. (10.1093/neuonc/nov044) 25838139 PMC5654354

[25] Eveslage M Calaminus G Warmuth-Metz M Kortmann RD Pohl F Timmermann B Schuhmann MU Flitsch J Faldum A & Muller HL. The postoperative quality of life in children and adolescents with craniopharyngioma. Deutsches Ärzteblatt International 2019 116 321–328. (10.3238/arztebl.2019.0321) 31219033 PMC6620763

[26] Beckhaus J Friedrich C Boekhoff S Calaminus G Bison B Eveslage M Timmermann B Flitsch J & Müller HL. Outcome after pediatric craniopharyngioma: the role of age at diagnosis and hypothalamic damage. European Journal of Endocrinology 2023 188. (10.1093/ejendo/lvad027) 36857103

[27] Kayadjanian N Hsu EA Wood AM & Carson DS. Caregiver burden and its relationship to health-related quality of life in craniopharyngioma survivors. Journal of Clinical Endocrinology and Metabolism 2023 109 e76–e87. (10.1210/clinem/dgad488) 37597173 PMC10735386

[28] Beckhaus J Friedrich C & Müller HL. Childhood-onset craniopharyngioma - a life-long family burden? Journal of Clinical Endocrinology and Metabolism 2024 109 e1404–e1405. (10.1210/clinem/dgad613) 37847153

