# Supplementary material for: Impact of parental body mass index at diagnosis on obesity in survivors of pediatric craniopharyngioma
Source: Endocr Connect. 2024 Jul 17;13(8):e240126. doi: 10.1530/EC-24-0126 (PMC11301543; doi:10.1530/EC-24-0126)
Supplement: Supplementary Material [file supplementary_material.pdf]

Table S1: Odds ratios from univariable logistic regression of patients' obesity with craniopharyngioma (CP) at last visit on categorical variable of parental overweight (>25 kg/m² BMI) vs. parental normal weight (>25 kg/m² BMI) at CP diagnosis.

**Contrast**	**Unadjusted OR**	**Lower limit 95% CI**	**Upper limit 95% CI**
Both overweight *vs.* both normal weight	2.87	1.53	5.49
Both normal weight *vs.* only mother overweight	0.39	0.13	1.12
Both normal weight *vs.* only father normal weight	0.78	0.34	1.81
Only mother overweight *vs.* only father overweight	2.01	0.71	5.76
Only mother overweight *vs.* both overweight	0.90	0.31	2.56
Only father overweight *vs.* both overweight	0.44	0.19	1.01

Abbreviations: CP, craniopharyngioma; BMI, body mass index; OR, odds ratio; CI, confidence interval.

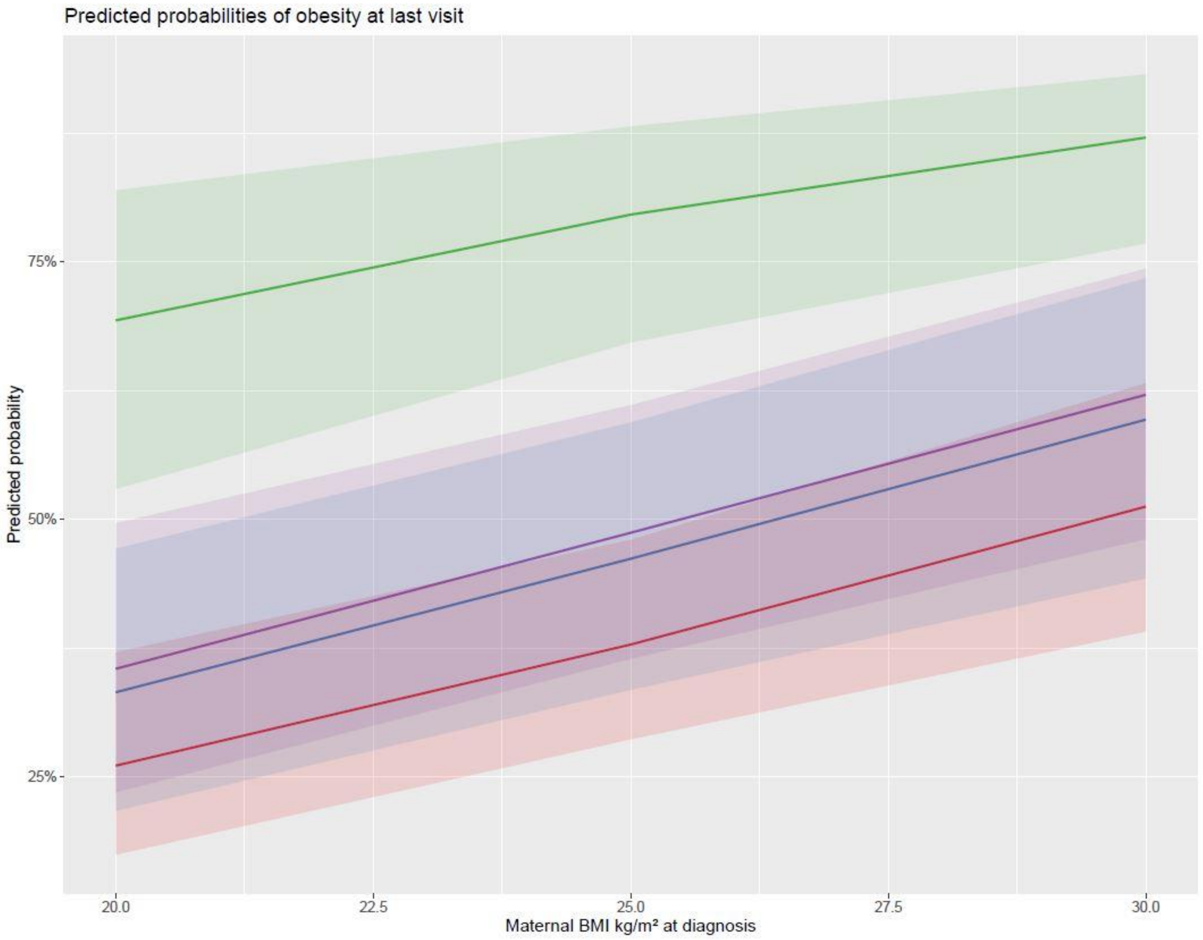

Figure S1: Marginal effect plot showing the probability of obesity in patients with childhood-onset craniopharyngioma (CP) at last visit for different values of maternal body mass index (BMI) at CP diagnosis in the four strata of hypothalamic damage (violet = HI I/II and HL = 0, red = no damage, blue = HI I/II and HL I, green = HI I/II and HL = II) adjusted additionally for follow-up, based on multivariable logistic regression model (Figure 2B).

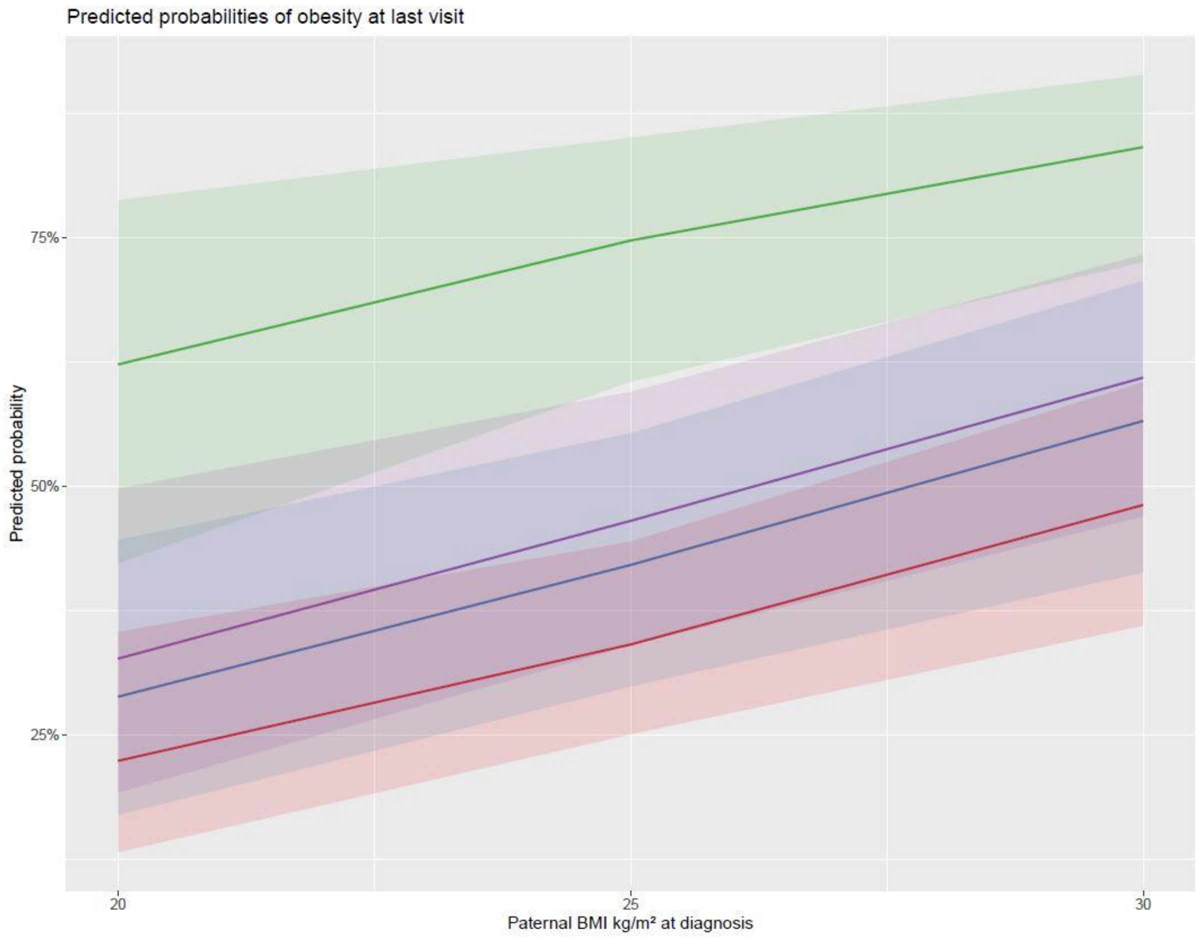

Figure S2: Marginal effect plot showing the probability of obesity in patients with childhood-onset craniopharyngioma (CP) at last visit for different values of paternal body mass index (BMI) at CP diagnosis in the four strata of hypothalamic damage (violet = HI I/II and HL = 0, red = no damage, blue = HI I/II and HL I, green = HI I/II and HL = II) adjusted additionally for follow-up, based on multivariable logistic regression model (Figure 2C).
